# Anti-sulfatide antibody-related Guillain–Barré syndrome presenting with overlapping syndromes or severe pyramidal tract damage: a case report and literature review

**DOI:** 10.3389/fneur.2024.1360164

**Published:** 2024-04-09

**Authors:** Xiaotian Ji, Jiaqian Zhu, Lujiang Li, Xiaodan Yang, Shaolong Zhou, Liming Cao

**Affiliations:** ^1^Department of Neurology, Sanya People’s Hospital, Sanya, China; ^2^School of Medicine, Shenzhen University, Shenzhen, China; ^3^Department of Neurology, Shenzhen Second People’s Hospital, Shenzhen, China; ^4^Department of Neurology, The First Affiliated Hospital of Shenzhen University, Shenzhen, China; ^5^Hunan Provincial Key Laboratory of the Research and Development of Novel Pharmaceutical Preparations, Changsha Medical University, Changsha, China

**Keywords:** anti-sulfatide antibody, Guillain–Barré syndrome, Miller–Fisher syndrome, Bickerstaff brainstem encephalitis, clinical characteristics, pyramidal tract damage, case report

## Abstract

**Introduction:**

Anti-sulfatide antibodies are key biomarkers for the diagnosis of Guillain–Barré syndrome (GBS). However, case reports on anti-sulfatide antibody-related GBS are rare, particularly for atypical cases.

**Case description, case 1:**

A 63 years-old man presented with limb numbness and diplopia persisting for 2 weeks, with marked deterioration over the previous 4 days. His medical history included cerebral infarction, diabetes, and coronary atherosclerotic cardiomyopathy. Physical examination revealed limited movement in his left eye and diminished sensation in his extremities. Initial treatments included antiplatelet agents, cholesterol-lowering drugs, hypoglycemic agents, and medications to improve cerebral circulation. Despite this, his condition worsened, resulting in bilateral facial paralysis, delirium, ataxia, and decreased lower limb muscle strength. Treatment with intravenous high-dose immunoglobulin and dexamethasone resulted in gradual improvement. A 1 month follow-up revealed significant neurological sequelae.

**Case description, case 2:**

A 53 years-old woman was admitted for adenomyosis and subsequently experienced sudden limb weakness, numbness, and pain that progressively worsened, presenting with diminished sensation and muscle strength in all limbs. High-dose intravenous immunoglobulin, vitamin B1, and mecobalamin were administered. At the 1 month follow-up, the patient still experienced limb numbness and difficulty walking. In both patients, albuminocytologic dissociation was found on cerebrospinal fluid (CSF) analysis, positive anti-sulfatide antibodies were detected in the CSF, and electromyography indicated peripheral nerve damage.

**Conclusion:**

Anti-sulfatide antibody-related GBS can present with Miller–Fisher syndrome, brainstem encephalitis, or a combination of the two, along with severe pyramidal tract damage and residual neurological sequelae, thereby expanding the clinical profile of this GBS subtype. Anti-sulfatide antibodies are a crucial diagnostic biomarker. Further exploration of the pathophysiological mechanisms is necessary for precise treatment and improved prognosis.

## 1 Introduction

Guillain–Barré syndrome (GBS) is an immune-mediated acute inflammatory demyelinating peripheral neuropathy typically triggered by infection or other immune stimuli that induce an abnormal immune response against peripheral nerves and their spinal roots. The global incidence of GBS varies from 0.6 to 4 cases per 100,000 individuals (1). In a significant number of patients, GBS results in disability or even death, and there are notable geographical variations in its occurrence (2). Anti-sulfatide antibody-related GBS is marked by antibodies against sulfatide, which are thought to contribute to the syndrome by attacking nerve cells.

Sulfatide, a major lipid component of the myelin sheath, is predominantly found in myelinating cells such as oligodendrocytes and Schwann cells, as well as in the nodes of Ranvier and paranodal regions (3). It is crucial for maintaining the structure of the nerve myelin sheath and for regulating nerve impulses and information transmission (3). Sulfatide plays crucial roles in the nervous system, including in the maintenance of myelin, regulation of neural function, immune response, and neuroinflammation (3, 4). It is a multifunctional molecule essential for various biological processes in the nervous and immune systems, and its abnormal expression can lead to disease (5). Pestronk et al. (6) first suggested the role of anti-sulfatide antibodies and found that high serum titers were associated with idiopathic, axonal, predominantly sensory neuropathies. The binding pattern of anti-sulfatide antibodies to neural tissues is associated with the type of neuropathy. Anti-sulfatide/GQ1b IgG antibodies have been found in 14% of patients with GBS, indicating that these antibodies may serve as an important biomarker for GBS (7). Patients with elevated levels of anti-sulfatide IgM antibodies exhibit chronic, slowly progressive, distally pronounced, symmetric polyneuropathy with sensorimotor impairment, ataxia, hyporeflexia, and axonal involvement (8).

Case reports on anti-sulfatide antibody-related GBS—especially on atypical cases—are scarce. Furthermore, the clinical characteristics and management of this GBS subtype remain poorly understood, particularly in atypical presentations. We report two such cases and review the relevant literature to enhance disease management strategies.

## 2 Case description

### 2.1 Patient 1

A 63 years-old man was admitted to our hospital in October 2021 because of limb numbness and double vision that he had experienced for half a month; his symptoms had worsened over the prior 4 days. His medical history included cerebral infarction, gout, and lumbar disc herniation for 7 years and type 2 diabetes and coronary atherosclerotic cardiopathy for 4 years. The patient had been consuming alcohol for several decades.

Physical examination on admission revealed a blood pressure of 140/100 mmHg, limited abduction in the left eye, and hypoesthesia in the extremities. No other abnormal neurological signs were present.

Blood analysis indicated that the red and white blood cell counts and fasting blood glucose, creatinine, urea nitrogen, alanine aminotransferase, aspartate aminotransferase, total cholesterol, triglyceride, electrolyte, and myocardial enzyme levels were within normal limits. Serum uric acid (451 μmol/L, reference range: 208–428 μmol/L) and albumin levels (36.6 g/L, reference range: 40–55 g/L) were abnormal. Chest computed tomography revealed aortic and coronary calcifications; echocardiography showed reduced left ventricular diastolic function; carotid artery and abdominal ultrasound detected fatty liver disease, renal cysts, and prostatic hyperplasia; magnetic resonance imaging revealed multiple lacunar infarctions, leukoencephalopathy, and slight brain atrophy, and magnetic resonance angiography demonstrated bilateral posterior cerebral artery (P2 segment) stenosis and partial occlusion of the bilateral distal branches of the middle cerebral artery.

Electromyography on day 1 indicated multifocal peripheral neuropathy of the limbs (demyelination combined with axonal damage) affecting both the motor and sensory nerves. Particularly, nerve conduction velocity indicated prolonged distal latency of the detected motor nerve conduction, decreased compound muscle action potential (CMAP) amplitude, and slowed motor conduction velocity of the right ulnar and peroneal nerves. Sensory nerve action potentials of the upper limbs were not elicited, and the sensory nerve action potential amplitude of the left superficial peroneal nerve decreased. There was prolonged F-wave latency of the right ulnar and median nerves, and bilateral tibial nerve H-reflexes were not elicited. Cerebrospinal fluid (CSF) analysis on day 7 revealed no white blood cells, an elevated protein level (3.37 g/L, reference range: 0.15–0.45 g/L), and normal glucose and adenosine deaminase levels. Western blot analysis detected anti-sulfatide IgG antibodies (Jinyu Medical Laboratory, Guangzhou, China) in the serum and CSF on day 7. Autoimmune encephalitis antibodies (NMDA, AMPA1, AMPA2, LGI1, CASPR2, GABAB, IgLON5, DPPX, GlyR1, DRD2, GAD65, mGluR5, mGluR1, Neurexin-3α, GABAA, KLHL11, and ganglionic AChR) and demyelinating antibodies (AQP4, MOG, and GFAP) in the CSF were not detected via cell-based assay (Jinyu Medical Laboratory).

The patient initially received antiplatelet, cholesterol-lowering, glycemic, cerebral circulation-improving, and symptomatic treatments. On day 2, he developed diplopia, limited left-eye abduction, and right-sided peripheral facial paralysis. By day 4, his condition had deteriorated, and he presented with bilateral peripheral facial paralysis, restlessness, incoherent speech, abdominal distension, a positive heel-knee-shin test, a positive Romberg’s sign, and a muscle strength score of 4/5 in both lower limbs.

On day 3, he was administered intravenous immunoglobulin (IVIG) 27.5 g/days (0.4 mg/kg) for 5 days and intravenous dexamethasone (10 mg/days) for 5 days, after which his symptoms started to improve. Electromyography on day 34 revealed multifocal peripheral nerve demyelination, and the axonal damage in the limbs was more severe than that observed at the initial examination. The patient was discharged on day 43 with no mental abnormalities, improved facial paralysis and limb numbness, and an increased muscle strength score of 4^+^/5 in the lower limbs. A 1 month follow-up assessment revealed mild left-sided peripheral facial paralysis, slight numbness in all four limbs, and a minor decrease in muscle strength in both lower limbs.

### 2.2 Patient 2

A 53 years-old woman was admitted to the Department of Obstetrics and Gynecology in June 2023 due to adenomyosis. She had a history of an ectopic pregnancy. On admission, a physical examination revealed no abnormal neurological signs.

Blood tests revealed an elevated white blood cell count (12.67 × 10^9^/L, reference range: 4–10 × 10^9^/L), an increased neutrophil-lymphocyte ratio (78.2%, reference range: 40–75%), and a reduced hemoglobin level (103 g/L, reference range: 110–150 g/L). The results of the coagulation and liver and kidney function tests were normal. B-ultrasound revealed adenomyosis with complicated uterine adenomyomas and a right ovarian cyst. Abdominal ultrasonography revealed left upper ureteral dilatation and mild hydronephrosis. Brain computed tomography findings were unremarkable, whereas chest computed tomography showed fibrous foci in the inferior lobe of the right lung and the lingual lobe of the left lung.

On day 2, the patient developed limb weakness, numbness, and pain in both lower limbs. By day 3, she was unable to move freely, experienced numbness and pain in the palms and lower limbs, had a low-grade fever, and had poor mental health and sleep. The patient was transferred to the Department of Neurology after a neurology consultation. Further examination revealed slight lower abdominal tenderness, hypoesthesia in the extremities, slightly reduced touch sensation of the trunk, and severely decreased muscle strength (scores of 0–1/5 in the upper extremities and 1/5 in the lower extremities) with weakened muscle tone and tendon reflexes. The patient was diagnosed with suspected GBS.

CSF analysis revealed an elevated protein level (0.59 g/L, reference range: 0.15–0.45 g/L) as well as white blood cell counts and glucose, chloride, and lactate dehydrogenase levels within normal ranges. Western blot analysis detected anti-sulfatide IgG antibodies (Jinyu Medical Laboratory) in the CSF; IgG and IgM antibodies for anti-GM1, anti-GM2, anti-GM3, anti-GM4, anti-GD1a, anti-GD1b, anti-GD2, anti-GD3, anti-GT1a, anti-GT1b, anti-GQ1b, and anti-aquaporin-4 were not detected. Brain and cervical spine magnetic resonance imaging revealed mild leukoencephalopathy. Electromyography on day 10 showed multifocal peripheral neuropathy with limb involvement, characterized by significant axonal damage and accompanied by demyelination affecting both motor and sensory nerves. Nerve conduction velocity indicated that sensory nerve actional potentials in the bilateral superficial peroneal and right median nerves were not elicited; the distal latency of the right median nerve was prolonged; the CMAP amplitude was decreased, and the conduction velocity was slowed. The motor conduction CMAP amplitude of the right ulnar nerve was decreased, with a decrease of more than 50% upon stimulation at the elbow. The motor conduction CMAP amplitude in the left common peroneal nerve was decreased. The F-waves of the median and ulnar nerves in the upper limbs and the H-reflex of the right tibial nerve were not elicited.

The patient received IVIG 25 g/days (0.4 mg/kg) for 5 days, intramuscular vitamin B1 100 mg/days, oral vitamin B6, and mecobalamin on day 2. By day 20, her limb weakness and numbness improved, although she experienced pain in both lower limbs. On day 30, she was discharged with hypoesthesia at the terminals of all four limbs and decreased muscle strength in the upper (1^+^/5) and lower limbs (2^+^/5). A 1 month follow-up assessment revealed persistent hypoesthesia at the terminals of the extremities, slightly improved muscle strength in the upper (2^+^/5) and lower limbs (3^+^/5), and the presence of a Babinski sign.

## 3 Discussion

This report demonstrates that patients with anti-sulfatide antibody-related GBS present with a high degree of clinical heterogeneity, which can manifest as Miller–Fisher syndrome (MFS), Bickerstaff brainstem encephalitis (BBE), or severe pyramidal tract damage, deviating from the typical understanding. This study enhances our understanding of the clinical phenotype of this type of GBS. Furthermore, anti-sulfatide antibody-related GBS can result in significant residual neurological sequelae even after active treatment. This report suggests that clinicians should be attentive to atypical or severe GBS, as early recognition and active treatment may improve prognosis.

### 3.1 Pathogenesis of anti-sulfatide antibody-related GBS

The presence of anti-sulfatide antibodies in patients with GBS, particularly the high prevalence of sulfatide antibodies, suggests a connection with disease pathogenesis (9, 10). Sulfatides are recognized as natural ligands of the TLR4-MD-2 complex, highlighting their role in the immune response and inflammation (11). Although antibodies targeting sulfatides may be pivotal to GBS development (12), the precise pathogenic mechanisms remain elusive. It has been hypothesized that anti-sulfatide antibodies are autoantibodies that erroneously attack and impair the myelin sheath function, causing nerve damage. Their presence indicates an immune reaction against sulfatides in patients with GBS. A previous study (13) showed that anti-sulfatide antibody-related demyelinating neuropathies involve complement-mediated pathological changes. These antibodies can accumulate in the peripheral nerve axons, sensory nerve endings, and neurons of the dorsal root ganglia, resulting in sensory axonal peripheral neuropathy (14). Thus, anti-sulfatide antibodies in GBS indicate an immune attack on sulfatides, leading to inflammation and myelin sheath damage. Elevated anti-sulfatide IgM levels are strongly indicative of chronic, immune-mediated, predominantly demyelinating neuropathy, which may have diagnostic significance (15).

### 3.2 Clinical characteristics of anti-sulfatide antibody-related GBS

It is generally believed that the clinical characteristics of anti-sulfatide antibody-related GBS are as follows: (1) initial presentation of limb numbness and weakness with involvement of the sensorimotor system, especially sensory disorders; (2) demyelination mainly of the sensory nerve, seen on electromyography, with good overall prognosis (6, 16, 17). Nevertheless, patient 1 initially experienced diplopia and limb numbness, followed by bilateral facial paralysis and manifestations of encephalopathy (mental disorder, ataxia, and pyramidal tract damage), and finally had obvious residual neurological sequelae. This patient simultaneously presented with the characteristic clinical features of both MSF (18, 19) and BBE (20), which has rarely been reported in patients with anti-sulfatide antibody-related GBS. MFS is considered the most common variant of GBS and is characterized by the medical triad of ophthalmoplegia, ataxia, and areflexia (18, 19). BBE is characterized by the medical triad of ophthalmoplegia, ataxia, and encephalopathy (20). BBE and MFS form a continuous spectrum with variable central and peripheral nervous system involvement (21), and BBE can present with overlapping features of GBS (22). The overlap of clinical syndromes in patients with GBS is a multifaceted phenomenon influenced by immune responses (23), diverse neurological manifestations (24), and potential interactions with infectious agents (25). Anti-sulfatide antibodies exhibit several different tissue-binding patterns in the peripheral and central nervous systems. These differences may be related to variations in clinical neuropathy syndromes associated with apparently similar anti-sulfatide antibodies (14).

Patient 2 initially experienced limb pain and severe pyramidal tract damage. The cause of the limb pain may be demyelination of the sensory nerve. Pyramidal tract injury can be observed in GBS due to other causes (10). The Babinski sign was influenced by the type of lesion and the damage to the nerves around the lower limbs (26); however, it is rarely reported in GBS related to anti-sulfatide antibodies. Patients with GBS may experience mononuclear cell infiltration into the cranial nerves, spinal ganglia, and spinal nerve roots. This can disrupt the blood-brain barrier due to inflammatory cell infiltration, permitting inflammatory factors to access the central nervous system and affect the corticospinal tract (27). Our study revealed the diverse clinical manifestations of anti-sulfatide antibody-related GBS, enriching our understanding of its clinical phenotype.

### 3.3 Electromyographic findings

Typical electromyographic findings in GBS include slowed nerve conduction, reduced CMAP, prolonged distal motor latency, and conduction block. However, the current understanding of electromyographic findings in anti-sulfatide antibody-related GBS remains incomplete. In this study, electromyography revealed a combination of demyelination and axonal damage of the peripheral nerves, consistent with previous reports (17). In patient 2, there was clear and severe evidence of multifocal axonal damage, indicating a potentially poor prognosis (28). Furthermore, electromyography conducted on day 34 in patient 1 revealed more severe nerve damage than that on day 1, which was consistent with the observed clinical symptoms. Hence, initial electromyography can indicate the type and extent of neural damage, and follow-up electromyography can serve as an objective basis for assessing treatment efficacy and predicting outcomes. It should be noted that the abnormalities in the nerve conduction study results were the most obvious 2 weeks after onset. Previous studies have reported electrodiagnostic findings in patients with anti-sulfatide antibody-associated polyneuropathy, including axonal, demyelinating, and normal nerve physiology (8, 29), emphasizing the diverse electromyographic presentations in this context. Nerve conduction studies can help to diagnose GBS and distinguish between axonal damage and demyelination. A previous study (16) revealed that decreased motor nerve conduction velocity of the common peroneal nerve and increased abnormalities in the distal CMAP amplitude suggest a poor prognosis.

### 3.4 Diagnosis of anti-sulfatide antibody-related GBS

Diagnosing anti-sulfatide antibody-related GBS typically involves assessing symptoms and signs, supported by CSF and electromyography findings, as well as the results of anti-sulfatide antibody testing. The diagnosis of anti-sulfatide antibody-related GBS requires the confirmation of the presence of anti-sulfatide antibodies and meeting the diagnostic criteria for GBS. Our study showed that antibodies are important diagnostic markers. Studies (15, 30) showed that high titers of anti-sulfatide antibodies may be involved in the occurrence and development of GBS. High anti-sulfatide IgM titers are highly predictive of chronic dysimmunity and may have diagnostic relevance (15). In conclusion, anti-sulfatide antibodies can be used as a diagnostic tool for peripheral neuropathy.

### 3.5 Treatment of anti-sulfatide antibody-related GBS

The current treatment for anti-sulfatide antibody-related GBS is referred from the treatment for GBS, which includes immunomodulatory therapies and supportive care. Treatments for patients with anti-sulfatide neuropathy include intravenous immunoglobulin, plasma exchange, and cyclophosphamide (31, 32); however, their potential efficacy in the context of anti-sulfatide antibody-related GBS warrants further investigation. Immunotherapy should be administered as soon as possible after disease onset, and IVIG and plasma exchange are recommended for GBS (33). Experimental studies have shown that introducing anti-sulfatide antibodies into the body can lead to demyelination of the peripheral nerves, suggesting that these antibodies may play a damaging role in certain neuropathies (34). This finding implies that therapies designed to counteract the harmful effects of anti-sulfatide antibodies on nerve function may be effective in treating these neuropathies. However, further research and clinical trials are required to develop reliable treatment protocols for this particular form of GBS.

Our patients’ conditions deteriorated during hospitalization. The typical treatment strategy for progressive disease is administration of IVIG or plasma exchange as soon as possible. IVIG can be readministered based on individual situations. Despite receiving standard immunotherapy, approximately 20% of the patients with GBS die or experience persistent disability (35). Recovery from GBS can be a slow process that can take several weeks or months. A thorough understanding of the pathogenesis and implementation of targeted therapy is crucial for improving prognosis.

### 3.6 Limitations

This report has certain limitations. It only describes two cases representing a specific clinical phenotype. Our findings provide valuable information for disease management. However, larger multicenter studies with more patients are necessary to confirm that these findings are generalizable.

## 4 Conclusion

Anti-sulfatide antibody-related GBS can manifest as MFS, BBE, or a combination of the two, along with severe damage to the pyramidal tract and long-term neurological complications, thereby expanding the clinical profile of this GBS subtype. Electromyography revealed a mix of demyelination and axonal damage in the peripheral nerves, aiding in assessing the extent of nerve damage and the efficacy of treatment. Anti-sulfatide antibodies are a crucial diagnostic biomarker of GBS. Further research into the pathophysiological mechanisms of this GBS subtype is essential for accurate treatment and improved prognosis.

## Patient perspective

The two patients are satisfied with the diagnosis and treatment they received.

## Data availability statement

The original contributions presented in the study are included in the article/supplementary material, further inquiries can be directed to the corresponding author.

## Ethics statement

The studies involving humans were approved by the Ethics Review Board of the Sanya People’s Hospital (No. 2023-190-01). The studies were conducted in accordance with the local legislation and institutional requirements. The participants provided their written informed consent to participate in this study. Written informed consent was obtained from the individual(s) for the publication of any potentially identifiable images or data included in this article.

## Author contributions

XJ: Formal analysis, Visualization, Writing – original draft, Resources. JZ: Writing – original draft, Data curation, Formal analysis, Investigation. LL: Formal analysis, Investigation, Writing – original draft. XY: Formal analysis, Validation, Writing – original draft. SZ: Writing – review & editing, Data curation, Resources. LC: Data curation, Funding acquisition, Writing – review & editing, Formal analysis, Methodology.
